# Nothing to sneeze at – uptake of protective measures against an influenza pandemic by people with schizophrenia: willingness and perceived barriers

**DOI:** 10.1177/1039856218815748

**Published:** 2018-12-03

**Authors:** Paul A Maguire, Rebecca E Reay, Jeffrey CL Looi

**Affiliations:** Lecturer and Acting Co-Deputy Head, Academic Unit of Psychiatry and Addiction Medicine, Australian National University Medical School, Canberra, ACT, Australia; Senior Research Coordinator and Lecturer, Academic Unit of Psychiatry and Addiction Medicine, Australian National University Medical School, Canberra, ACT, Australia; Associate Professor and Acting Head, Academic Unit of Psychiatry and Addiction Medicine, Australian National University Medical School, Garran, ACT, and; Clinical Associate Professor, Department of Psychiatry, Royal Melbourne Hospital, Melbourne, VIC, and; Director, Research Centre for the Neurosciences of Ageing, Canberra Hospital, Canberra, ACT, Australia

**Keywords:** pandemic influenza, protective measures, willingness, perceived barriers, schizophrenia

## Abstract

**Objectives:**

To examine willingness to adopt protective behaviours, and perceived barriers, during a pandemic influenza, in people with schizophrenia.

**Methods:**

A cross-sectional study using a questionnaire was conducted exploring the responses of 71 adults with schizophrenia and 238 adults without schizophrenia attending a general practice setting, regarding willingness and perceived barriers to adopting protective measures against the 2009 swine influenza pandemic in Australia.

**Results:**

The majority of participants with schizophrenia reported that they would be at least moderately willing to be vaccinated (74.2%), isolate themselves (73.2%), wear a face mask (54.9%) and increase hand washing (88.6%). However, 71.8% were concerned about “catching” flu from vaccination. Predictors of willingness to adopt protective actions included self-efficacy (vaccination, face mask, isolation), perceived likelihood of contracting swine flu (vaccination), educational status (face mask) and perceived overall risk from swine flu (face mask). Key modifiable perceived barriers to adopting protective measures were identified, including cost and need for transport assistance for vaccination.

**Conclusions:**

People with schizophrenia report being generally willing to adopt protective measures, especially increased hand washing, during a pandemic influenza. Understanding perceived barriers may enable development of effective interventions to increase uptake of protective measures.

Pandemic influenza continues to pose a major public health threat in the 21^st^ century. Therefore, it is essential that health services are maximally prepared with a well-considered response plan. Health outcomes of a significant influenza outbreak are influenced by the behavioural response of the community. The adoption of protective measures by the public attenuates the spread of the virus, especially in the early stages of a pandemic. In particular, vaccination can significantly reduce the number of people infected, as well as hospitalization and mortality rates.^1^ However, because there are likely to be limitations in the supply and effectiveness of vaccines (and antiviral medication) in a pandemic influenza, non-pharmacological (infection control) measures also have an important role to play.^2^ Increased hand hygiene, isolation and wearing a face mask have been demonstrated to be effective in reducing transmission of respiratory viruses^3^ and their use during a pandemic influenza has been endorsed by expert opinion.^2,4,5^ Protective behaviour is linked to a person’s risk perceptions of the health threat as well as their evaluation of the potential benefits and risks associated with a given preventative action.^6^

People with a mental illness, including those with schizophrenia, have been shown to have significantly higher mortality rates from influenza and pneumonia compared with the general population, as well as a 50% excess hospitalization for influenza.^7^ However, there is a paucity of research investigating: what people with mental illness believe about protective measures; how willing they would be to adopt them; and what they perceive as barriers to their uptake, in the event of a significant outbreak of influenza.

This study was conducted in 2009 during the H1N109 (“swine flu”) outbreak in Australia, which the World Health Organization declared a global pandemic on 11 June 2009. The key aims of this study were to explore the willingness of people with schizophrenia to undertake protective measures against the 2009 swine influenza, as well as perceived barriers to adopting these measures.

## Methods

The following is a summary of the methods used in this study, which have been described elsewhere.^8^

### Participants

The sample has been described previously^8^ but can be summarized as follows. Within the Australian Capital Territory, a purposive stratified sample of 309 patients aged 18–65 (inclusive) was recruited from healthcare settings. This included 71 patients with schizophrenia (SCZ) from mental health care settings, as well as 238 adults from 13 general practice (GP) settings, none of whom had a diagnosis of schizophrenia. Participants with schizophrenia (excluding schizoaffective disorder) were recruited from both hospital (*n* = 12) and community settings (*n* = 59). A diagnosis of schizophrenia was confirmed by the patient’s treating psychiatrist. Approval was obtained from ACT Health and Australian National University ethics committees. Written consent was obtained from all participants.

### Measures

Participants were invited to complete a questionnaire that included items examining their willingness to undergo protective measures during a pandemic influenza in Australia, and perceived barriers to adopting these measures (Table 1).

**Table 1.** Questionnaire items

**(1) Socio-demographic characteristics** Age, gender, highest level of education, employment status, children in the household, living alone, and non-English language spoken in the household. **(2) Willingness to adopt protective measures** *In the case of an emergency such as an influenza pandemic, government authorities might request cooperation from the public in a number of ways. Please indicate*: *How willing would you be to* … [each protective action – receive a vaccination, isolate yourself from others, wear a face mask, wash your hands more frequently *–* enquired about individually] Response options included: 1 = Not at all willing; 2 = A little willing; 3 = Moderately willing; 4 = Very willing; and 5 = Extremely willing A “Don’t know” response option was included for vaccination. **(3) Perceived effectiveness of each protective measure** *How effective do you think…*[each protective measure]…*would be in preventing you from catching influenza during a pandemic outbreak?* Participants responded on a five-point scale ranging from “Not at all effective” to “Extremely effective”. **(4) Self-efficacy** Self-efficacy facilitates adaptive heath behaviours and is included as a component of health behaviour models such as the Health Belief Model, e.g. seasonal uptake of vaccination is strongly associated with self-efficacy. Even if an individual believes the risk benefit profile of a protective action is favourable, there still appears to be a need for the belief that they can actually go ahead and carry out this action. *How confident are you that once you decided to* …[each protective action]…*you would be able to actually go each and do this?* **(5) Risk perception and feelings of vulnerability** *Overall, what do you see as your risk from human swine influenza if you took no protective actions?* How vulnerable does it make you feel knowing that there is a global influenza pandemic? Participants responded to each of these questions on a five-point scale ranging from “No risk at all” to “An extreme risk”, and “Not at all vulnerable” to “Extremely vulnerable”, respectively. Similarly, participants were asked about they perceive their likelihood of contracting swine flu and how serious this would be for them if they did. In addition, knowledge of the disease experience was ascertained by asking, “Have you or someone close to you ever suffered from a serious influenza in the past?” **(6) Perceived barriers** Participants were asked an open-ended question to explore what they viewed as potential barriers to carrying each of the four protective measures: *What might be difficult for you about …*[each protective action] …? *Please name three things.* **(7) Single-item Self-Rated Health Question (SRHQ) and 10-item Kessler Psychological Distress** Scale (K-10) Because risks associated with influenza are heightened in those with concurrent medical illness, and this may impact on willingness to undertake protective measures, an SRHQ^9^ was included in the questionnaire. Given that psychological distress may impact on willingness to take up protective measures, a K-10 was also included.

### Statistical analyses

All analyses were carried out using SPSS version 22. Within-group analyses using logistic regression were performed to examine for significant predictors of willingness to take protective actions. Odds ratios were obtained after responses were dichotomized into: **(1)** “Not at all” or “A little”, and **(2)** “Moderately”, “Very” or “Extremely”. For perceived barriers to protective actions, responses from the SCZ and GP groups were assigned to identified themes for each protective action, including a “No difficulty” category. The proportion of responders in each theme category was calculated.

## Results

### Demographics

The mean age in the SCZ group was 36.1 years (SD 9.7) and 36.6 years (SD 14.1) in the GP group (Table 2). There was no statistically significant difference between the SCZ and GP groups for age. However, significant differences were present for gender, employment status, living alone, children in the household, and highest level of education.

**Table 2.** Socio-demographic characteristics

	SCZ	GP	Statistic^a^
	(*n* = 71)	(*n* = 238)	
**Age**			*t* = 0.36, *p* = 0.72
Mean	36.1	36.6	
SD	9.7	14.0	
Range	43 (19–62)	47 (18–65)	
**Gender**			χ^2^ = 28.9, *p <* 0.01
Male	70%	34%	
**Highest level of education**			**χ** ^2^ **= 50.7, *p <* 0.01**
None	11.3%	1.7%	
Year 10 certificate	31.0%	15.1%	
Year 12 certificate	25.4%	20.2%	
TAFE certificate/diploma	23.9%	14.3%	
University degree	8.5%	48.7%	
**Currently employed**	21.1%	91.7%	χ^2^ = 50.3, *p <* 0.01
**Children in household**	5.6%	43.7%	χ^2^ = 33.2, *p <* 0.01
**Lives alone**	33.8%	12.2%	χ^2^ = 16.5, *p <* 0.01
**Non-English language spoken in the household**	20.0%	22.5%	χ^2^ = 0.07, *p* = 0.79

Bold indicates statistical significance (*p* < 0.05).

GP: general practice; SCZ: schizophrenia; SD: standard deviation; TAFE: Technical And Further Education.

a Statistic includes: independent-samples *t*-test; chi-square test.

### Willingness

#### Vaccination

In the SCZ group, 74.2% of participants were moderately, very or extremely willing to receive a vaccination compared with 80.1% in the GP group (Table 3). Within-group analysis (Table 4) revealed that, for participants in the SCZ group, predictors of increased willingness to receive a flu vaccination included self-efficacy and perceived own likelihood of contracting swine influenza. In the GP group, predictors of increased willingness to be vaccinated included perceived effectiveness of vaccination and self-efficacy. Those in the GP group who perceived a substantive risk of adverse reaction were less likely to be willing to receive a vaccination.

**Table 3.** Comparison between SCZ and GP groups for protective measures against pandemic influenza

Precautionary measure^a^	SCZ (%)	GP (%)	AOR (95% CI)	*p* value
	(*n* = 71)	(*n* = 238)		
**Vaccination**				
Willing to receive	74.3	80.1	**0.41 (0.19–0.88)**	**0.02**
Perceived as effective	86.6	75.3	1.63 (0.69**–**3.86)	0.27
Perceived as risky for adverse reaction	38.7	27.5	**2.17 (1.03–4.56)**	**0.04**
Concern about “catching” flu from vaccination	71.8	50.2	**2.19 (1.48–3.25)**	**0.02**
Self-efficacy	85.5	76.5	0.72 (0.44**–**1.17)	0.43
**Isolation**				
Willing to be isolated	73.2	86.1	**0.41 (0.25–0.65)**	**0.03**
Perceived as effective	69.7	80.9	0.52 (0.33**–**0.81)	0.09
Self-efficacy	61.8	72.6	**0.44 (0.29–0.66)**	**0.02**
**Face mask**				
Willing to wear	54.9	61.6	0.44 (0.49**–**1.17)	0.40
Perceived as effective	45.5	57.7	0.52 (0.27**–**1.01)	0.05
Self-efficacy	63.2	66.0	0.90 (0.45**–**1.79)	0.77
**Hand washing**				
Willing to wash hands more frequently	88.6	93.2	0.78 (0.25**–**2.41)	0.58
Perceived as effective	77.3	85.6	0.62 (0.27**–**1.41)	0.17

a At least a moderate amount of that variable (e.g. *willingness to receive vaccination* denotes reporting being moderately, very or extremely willing) except for *Concern about “catching” flu from a vaccination*, which denotes any degree of concern at all.

Bold indicates statistically significant results (*p* < 0.05).

AOR: adjusted odds ratio (adjusted for age, gender, employment status, level of education, living alone, children in the household, non-English language spoken at home, week of participation, self-rated health, previous influenza experience and K10 total score); CI: confidence interval; GP: general practice; SCZ: schizophrenia.

**Table 4.** Predictors of willingness to adopt protective measures: within-group multiple logistic regression (Exp(B) with *p* values).

	Vaccination		Isolation		Face mask		Increased hand washing	
	SCZ (*n* = 71)	GP (*n* = 238)	SCZ (*n* = 71)	GP (*n* = 238)	SCZ (*n* = 71)	GP (*n* = 238)	SCZ (*n* = 71)	GP (*n* = 238)
**Predictor**								
**Age**	1.01 (0.80)	**0.96 (0.01)**	1.16 (0.05)	**1.05 (0.02)**	1.10 (0.05)	1.02 (0.27)	1.03 (0.57)	0.98 (0.57)
**Gender (male)**	1.76 (0.58)	1.09 (0.87)	4.54 (0.19)	(0.90 (0.84)	1.75 (0.48)	0.81 (0.61)	2.49 (0.48)	0.24 (0.05)
**Employed**	1.08 (0.95)	1.12 (0.84)	0.68 (0.73)	(0.42 (0.16)	1.14 (0.88)	1.29 (0.59)	0.34 (0.38)	1.38 (0.65)
**Highest level of education**	-	-	-	**40.00** ^a^ **(0.03)**	**0.02**^b^ **(0.02)** 0.02^a^ (0.03)	-	-	-
**Self-rated general health**	1.38 (0.64)	1.04 (0.89)	0.36 (0.22)	0.75 (0.33)	1.41 (0.51)	**1.75 (0.02)**	0.79 (0.76)	**0.33 (0.02)**
**Perceived effectiveness**	1.67 (0.29)	**3.66 (< 0.01)**	**5.23 (0.01)**	1.25 (0.29)	1.27 (0.58)	**2.48 (< 0.01)**	2.98 (0.09)	**3.68 (< 0.01)**
**Perceived risk of adverse reaction**	0.55 (0.11)	**0.58 (0.01)**	nd	nd	nd	nd	nd	nd
**Self-efficacy**	**3.44 (0.04)**	**1.72 (< 0.01)**	**4.89 (0.01)**	**2.18 (< 0.01)**	**2.43 (0.02)**	**3.01 (< 0.01)**	nd	nd
**Perceived likelihood of self contracting swine flu**	**3.48 (0.04)**	0.91 (0.69)	1.53 (0.53)	0.83 (0.41)	0.59 (0.22)	1.41 (0.22)	0.31 (0.12)	0.92 (0.76)
**Perceived seriousness of self contracting swine flu**	2.16 (0.16)	1.13 (0.64)	0.83 (0.77)	1.31 (0.28)	0.62 (0.32)	1.31 (0.22)	1.65 (0.48)	0.99 (0.98)
**Feeling vulnerable to swine flu**	1.07 (0.88)	1.35 (0.29)	0.53 (0.25)	0.83 (0.47)	0.93 (0.85)	1.11 (0.65)	3.34 (0.16)	0.61 (0.20)
**Perceived overall risk to self from swine flu**	0.76 (0.58)	1.05 (0.88)	4.82 (0.08)	1.35 (0.33)	**5.61 (0.01)**	1.27 (0.39)	1.72 (0.52)	**3.65 (0.01)**

Bold indicates statistically significant results (*p* < 0.05); Exp(B), exponential of regression coefficient B; nd, no data.

a *University degree* compared with *no educational attainment* (using dummy variables to represent highest educational attainment, reference group = “*None*”).

b *Year 10 certificate* compared with *no educational attainment* (using dummy variables to represent highest educational attainment, reference group = “*None*”).

GP: general practice; SCZ: schizophrenia.

### Social isolation

In the SCZ group, 73.2% of participants were at least moderately willing to isolate themselves from others compared with 86.1% in the GP group (Table 3). Within-group analysis (Table 4) showed that in the SCZ group, positive predictors for willingness to be isolated included perceived effectiveness of isolation and self-efficacy. In the GP group, educational attainment and self-efficacy were positive predictors.

### Facial mask

In the SCZ group, 54.9% of participants were at least moderately willing to wear a face mask, compared with 61.6% in the GP group (Table 3). Of all the precautionary measures examined in both groups, wearing a face mask was the least likely to be adhered to and the most likely to be viewed as ineffective or minimally effective. Within-group analysis (Table 4) revealed that in the SCZ group, positive predictors of willingness to wear a face mask included self-efficacy and perceived overall risk from swine flu. Negative predictors included a university degree compared with no educational attainment, and a Year 10 Certificate compared with no educational attainment. In the GP group, self-efficacy, perceived effectiveness, and higher self-rated general health were positive predictors of willingness to wear a facial mask.

### Hand washing

There were no differences between the SCZ and GP groups in terms of being at least moderately willing to increase hand washing in the event of an Australian pandemic influenza (Table 3). Approximately 90% of participants in both groups were willing to engage in this simple but important protective measure. In addition, over three-quarters of people in each group evaluated increased hand washing as an effective preventative action. Perceived effectiveness and perceived substantive overall risk from swine flu increased the likelihood of people in the GP group being willing to increase hand washing, whereas higher self-rated general health reduced the likelihood (Table 4).

#### Between-group differences

Despite the majority of people in both groups being willing to adopt all four protective measures, logistic regression analysis revealed that people with schizophrenia were less willing to receive a vaccination and to isolate themselves compared with those in the GP group, had less self-efficacy for isolation, and were more likely to perceive vaccination as risky for an adverse reaction, including having concerns about “catching” flu from it (Table 3).

### Perceived barriers

**Figure 1.**
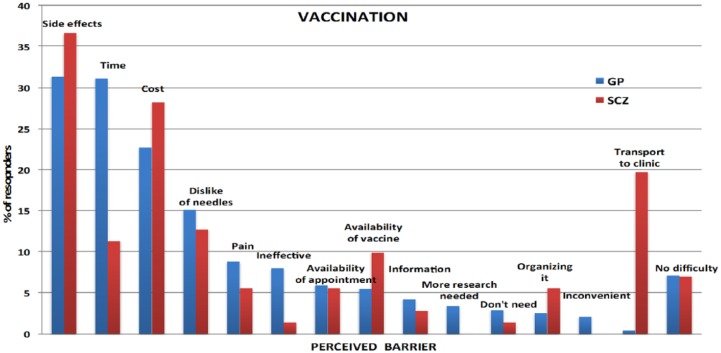
Perceived barriers to vaccination.

**Figure 2.**
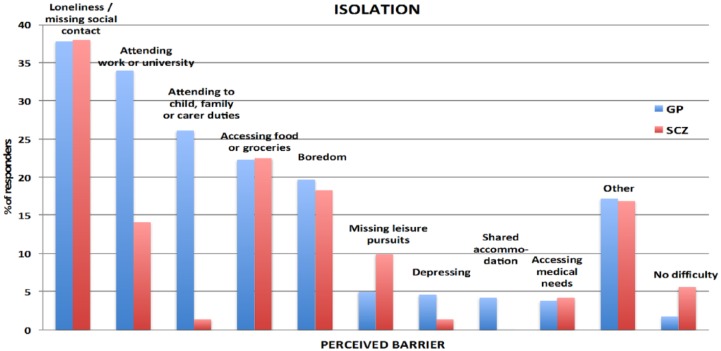
Perceived barriers to isolating oneself.

**Figure 3.**
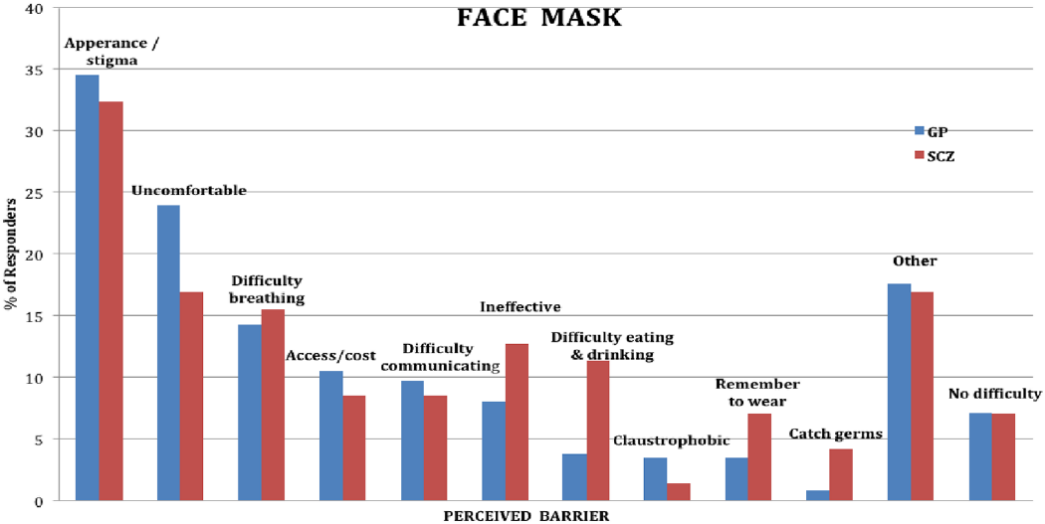
Perceived barriers to wearing a face mask.

**Figure 4.**
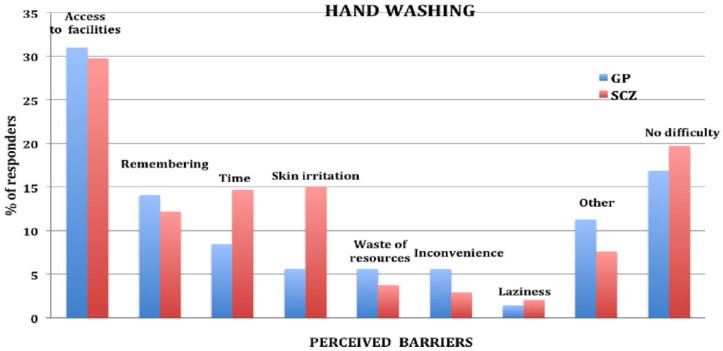
Perceived barriers to increased hand washing.

Perceived barriers for each protective measure are shown in Figures 1–4. For people with schizophrenia the main barriers are summarized in Table 5.

**Table 5.** Summary of commonest perceived barriers for people with schizophrenia

**Vaccination**	• Concern about side effects • Cost • Transport to a clinic (to receive vaccination)
**Isolation**	• Loneliness / missing social contact • Accessing food and groceries • Boredom
**Face mask**	• Appearance / stigma • Uncomfortable • Difficulty breathing
**Increased hand washing**	• Access to hand washing facilities (e.g. basin) • Skin irritation • Time

## Discussion

The results indicate that most people with schizophrenia attending public mental health services and GP attendees are willing to take protective action against pandemic influenza. In both groups, increased hand washing was the most accepted measure and wearing a face mask the least accepted. Hand washing as a simple, inexpensive and effective protective measure should be a core focus in public health messaging during a pandemic.

Significant perceived barriers exist for each of the protective measures for people with schizophrenia and in the general population. Although there are similarities between the two groups in how they perceive barriers, there are also substantive differences, which are likely to reflect *socio-demographic* disparities between the groups. Being unemployed, living alone, not having children in the household and having lower educational attainment are all likely to impact on perceived barriers. Barriers frequently identified by people without schizophrenia, such as needing to attend to child, family and carer duties or to attend work or university, were considerably lower in frequency for people with schizophrenia, whereas cost of purchasing a vaccination and difficulties with transport to a health facility to have it administered were significantly greater. Assisting people in overcoming perceived barriers may increase self-efficacy (found to be a strong predictor of willingness) and the uptake of protective measures during an influenza pandemic. A key strategy for increased hand hygiene during a pandemic may be education and encouragement in using antiseptic gels, given that lack of washing facilities was the most frequently cited barrier for increased hand washing in both groups. As people with schizophrenia were generally willing to receive a vaccination, assistance with overcoming perceived barriers could form part of pandemic influenza response planning. These measures could include: funding the vaccine, facilitating its administration (home visit or provision of transport), and education and correction of misconceptions about contracting influenza from a vaccination.

Limitations of this study include relatively small sample size, self-selection bias and clustering sampling, and the cross-sectional nature of the study.

## Conclusion

People with schizophrenia report being generally willing to adopt protective measures, especially increased hand washing, during a pandemic influenza. Understanding and further investigating perceived barriers may enable development of effective interventions to increase uptake of protective measures.

## References

[1] Nuno M Chowell G Gumel AB Assessing the role of basic control measures, antivirals and vaccines in curtailing pandemic influenza: Scenarios for the US, UK and The Netherlands. J R Soc Interface 2007; 4: 505–521. 17251132 10.1098/rsif.2006.0186 PMC2373400

[2] Aledort JE Lurie N Wasserman J Non-pharmaceutical public health intervention for pandemic influenza: An evaluation of the evidence base. BMC Public Health 2007; 7: 208. 17697389 10.1186/1471-2458-7-208 PMC2040158

[3] Jefferson T Jones M Doshi P Physical interventions to interrupt or reduce the spread of respiratory viruses. Cochrane Database Syst Rev 2011; CD006207. 10.1002/14651858.CD006207.pub4 PMC6993921 21735402

[4] Centres for Disease Control and Prevention. Novel H1N1 Flu Situation Update, http://www.cdc.gov/h1n1flu/updates/072409.htm (2009, accessed on 22 February 2011).

[5] Centres for Disease Control and Prevention. Interim Recommendations for Facemask and Respirator Use to Reduce 2009 Influenza A (H1N1) Virus Transmission, CDC, http://www.cdc.gov/h1n1flu/mask.htm (2009, accessed on 22 February 2011).

[6] Brewer NT Chapman GB Gibbons FX Meta-analysis of the relationship between risk perception and health behaviour: The example of vaccination. Health Psychol 2007; 26: 136–145. 17385964 10.1037/0278-6133.26.2.136

[7] Lawrence D Holman CDJ Jablensky AV (2001) Preventable Physical Illness in People with Mental Illness. Perth: The University of Western Australia.

[8] Maguire PA Reay RE Looi JCL et al Neither the internist nor the Internet: Use of and trust in health information sources in people with schizophrenia. Aust N Z J Psychiatry 2011; 45: 489–497. 21563868 10.3109/00048674.2011.570308

[9] DeSalvo KB Bloser N Reynolds K Mortality prediction with a single general self-rated health question: A meta-analysis. J Gen Intern Med 2005; 20: 267–275. 16336622 10.1111/j.1525-1497.2005.00291.x PMC1828094

